# AKAP12 Supports Blood-Brain Barrier Integrity against Ischemic Stroke

**DOI:** 10.3390/ijms21239078

**Published:** 2020-11-28

**Authors:** Ji Hae Seo, Takakuni Maki, Nobukazu Miyamoto, Yoon Kyong Choi, Kelly K. Chung, Gen Hamanaka, Ji Hyun Park, Emiri T. Mandeville, Hajime Takase, Kazuhide Hayakawa, Josephine Lok, Irwin H. Gelman, Kyu-Won Kim, Eng H. Lo, Ken Arai

**Affiliations:** 1 Neuroprotection Research Laboratory, Departments of Radiology and Neurology, Massachusetts General Hospital and Harvard Medical School, Charlestown, Boston, MA 02129, USA; seojh@kmu.ac.kr (J.H.S.); harutoma@kuhp.kyoto-u.ac.jp (T.M.); nobu-m@juntendo.ac.jp (N.M.); ykchoi@konkuk.ac.kr (Y.K.C.); kkchung@mgh.harvard.edu (K.K.C.); ghamanaka@mgh.harvard.edu (G.H.); JPARK79@mgh.harvard.edu (J.H.P.); ETMANDEVILLE@mgh.harvard.edu (E.T.M.); htakase@yokohama-cu.ac.jp (H.T.); khayakawa1@partners.org (K.H.); JLOK1@mgh.harvard.edu (J.L.); 2 Depart of Biochemistry, School of Medicine, Keimyung University, Daegu 42601, Korea; 3 Department of Cancer Genetics and Genomics, Roswell Park Comprehensive Cancer Center, Buffalo, NY 14203, USA; Irwin.gelman@roswellpark.org; 4 College of Pharmacy and Research Institute of Pharmaceutical Sciences, Seoul National University, Seoul 08826, Korea; qwonkim@snu.ac.kr

**Keywords:** A-kinase anchor protein 12, Rho kinase, stroke, endothelial cell, blood-brain barrier

## Abstract

A-kinase anchor protein 12 (AKAP12) is a scaffolding protein that associates with intracellular molecules to regulate multiple signal transductions. Although the roles of AKAP12 in the central nervous system are still relatively understudied, it was previously shown that AKAP12 regulates blood-retinal barrier formation. In this study, we asked whether AKAP12 also supports the function and integrity of the blood-brain barrier (BBB). In a mouse model of focal ischemia, the expression level of AKAP12 in cerebral endothelial cells was upregulated during the acute phase of stroke. Also, in cultured cerebral endothelial cells, oxygen-glucose deprivation induced the upregulation of AKAP12. When AKAP12 expression was suppressed by an siRNA approach in cultured endothelial cells, endothelial permeability was increased along with the dysregulation of ZO-1/Claudin 5 expression. In addition, the loss of AKAP12 expression caused an upregulation/activation of the Rho kinase pathway, and treatment of Rho kinase inhibitor Y-27632 mitigated the increase of endothelial permeability in AKAP12-deficient endothelial cell cultures. These in vitro findings were confirmed by our in vivo experiments using *Akap12* knockout mice. Compared to wild-type mice, *Akap12* knockout mice showed a larger extent of BBB damage after stroke. However, the inhibition of rho kinase by Y-27632 tightened the BBB in *Akap12* knockout mice. These data may suggest that endogenous AKAP12 works to alleviate the damage and dysfunction of the BBB caused by ischemic stress. Therefore, the AKAP12-rho-kinase signaling pathway represents a novel therapeutic target for stroke.

## 1. Introduction

Stroke is one of the most serious diseases, and the therapeutic options for stroke patients remain limited to reperfusion with a tissue plasminogen activator or a mechanical catheter device [1,2]. Over the past decades, advances in molecular and cellular biology have defined many potential mechanisms and targets for stroke. However, it is still difficult to translate these gains in basic knowledge into real clinical applications [3,4]. During the acute phase of stroke, several deleterious cascades are activated, but some signaling pathways may work to suppress these cascades through compensatory responses [5,6]. Nevertheless, the research for pro-survival signaling during the acute phase of stroke is still understudied. To develop an effective approach for brain protection against stroke, we may need to pay more attention to these compensatory signals.

AKAP12 is a scaffolding protein that associates with protein kinase A (PKA) and protein kinase C (PKC) to regulate signal transduction, such as PKA-CREB pro-survival signaling [7,8]. The function/role of AKAP12 has been extensively examined in cancer research [9], but its roles in the CNS are still largely unknown. However, some recent pre-clinical studies have highlighted the importance of the roles of AKAP12 in the brain. For example, AKAP12 regulates oligodendrocyte generation in cerebral white matter in mice [10]. In addition, in a mouse stroke model, AKAP12-positive cells in fibrotic scars restrict immune cells from entering the site of injury, which enhances CNS recovery [11]. Notably, AKAP12 is known to regulate the formation of the blood-retinal barrier (BRB) [12], which prevents the passage of certain substances from proceeding from the circulating blood into the retina. In the brain, the blood-brain barrier (BBB) plays similar roles in regulating the passage of molecules between the circulating blood and brain parenchyma [13,14,15]. BBB damage/dysfunction is one of the major hallmarks of stroke, which causes edema formation, and in a rat stroke model, it is implied that AKAP12 expression was negatively correlated with edema formation after stroke [16]. Therefore, in this study we used in vitro and in vivo systems to examine the roles of AKAP12 in BBB function during the acute phase of stroke.

## 2. Results

**Figure 1:**
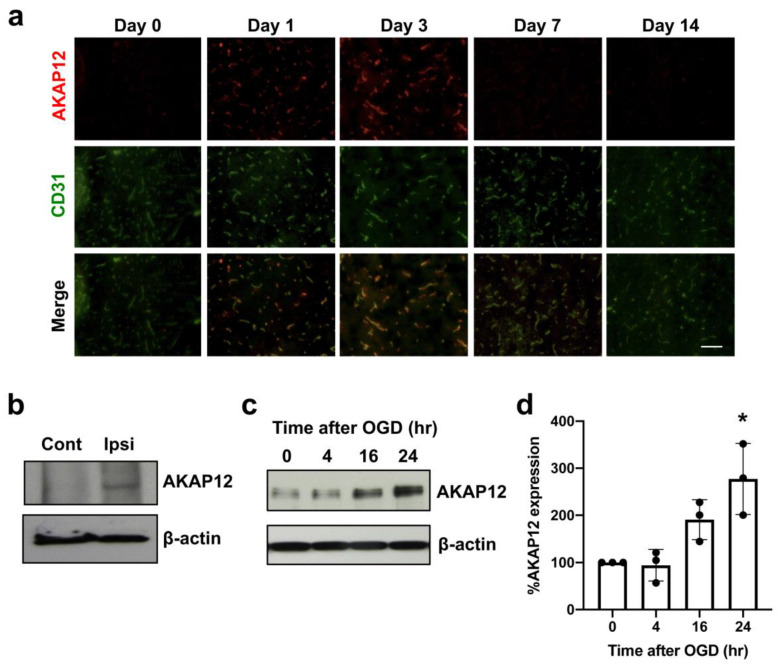
**AKAP12 expression after stroke:** (**a**) Double-staining of AKAP12 (red) with an endothelial marker CD31 (green) showed that AKAP12 was expressed in or around blood vessels, and its expression level transiently increased after stroke. Scale bar = 50 μm. (**b**) Western blot using endothelial fractions from stroke mice confirmed that compared to the contralateral hemisphere (Cont), AKAP12 level was elevated in the brain endothelium of the ipsilateral hemisphere (Ipsi) at 3 days after stroke. (**c**,**d**) In HBMECs, OGD/Reoxygenation stimulation, an in vitro model to mimic in vivo cerebral ischemia-reperfusion, induced an upregulation of AKAP12. Mean ± SD of *n* = 3. * *p* < 0.05 vs. control (0 hr after OGD). Kruskal-Wallis test followed by post-hoc Dunn’s multiple comparison test.

**Figure 2:**
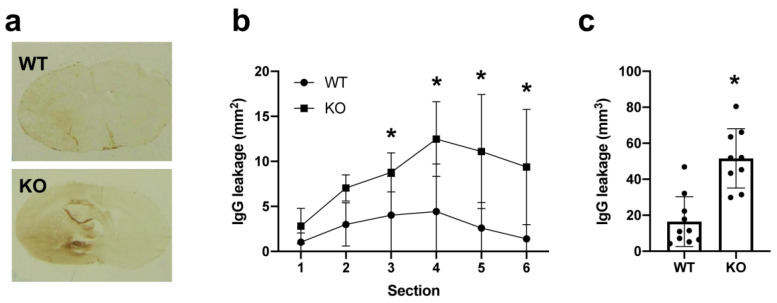
**BBB leakage after stroke in mice:** Akap12 KO mice or wild type (WT) mice were subjected to transient MCAO, and 3 days later, they were sacrificed for IgG staining. In WT mice, more IgG leakage was observed in the ipsilateral side compared to the contralateral side. In addition, KO mice showed exacerbated BBB damage, as assessed by IgG positive area. (**a**) Representative IgG staining images. (**b**) Quantitative results of IgG leakage in each brain section. Mean + SD of *n* = 9–10 per group. * *p* < 0.05 vs. WT for individual section. Two-way ANOVA followed by post-hoc Sidak’s multiple comparisons test. (**c**) Quantitative results of IgG leakage. Mean + SD of *n* = 9–10 per group. * *p* < 0.05 vs. WT. Mann-Whitney test.

We first examined expression patterns of AKAP12 after stroke in mice. During the acute phase of stroke, AKAP12 expression transiently increased in the affected region (Figure 1a). Double-staining with endothelial marker CD31 indicated that AKAP12 expression was mostly in or around cerebral vasculature (Figure 1a). Western blotting with endothelial fractions (e.g., isolated brain microvessels) from non-ischemic or ischemic regions showed that endothelial-AKAP12 expression indeed increased after stroke (Figure 1b). Furthermore, in vitro experiments using cultured cerebral endothelial cells (HBMECs) confirmed that AKAP12 expression was upregulated after in-vitro stroke conditions (e.g., oxygen-glucose deprivation: OGD) (Figure 1c–d). We then investigated whether AKAP12 has a supportive or detrimental role for BBB tightness after stroke by comparing *Akap12* knockout mice to wild type mice. As expected, on day 3 after stroke, the ipsilateral side (ischemic side) exhibited more IgG leakage (a marker for BBB damage) compared to the contralateral side (non-ischemic side) in wild type mice (Figure 2). Notably, the leakage of IgG caused by ischemic stroke was exacerbated in *Akap12* knockout mice (Figure 2), suggesting the supportive role of endogenous AKAP12 in BBB tightness against ischemic stress.

**Figure 3:**
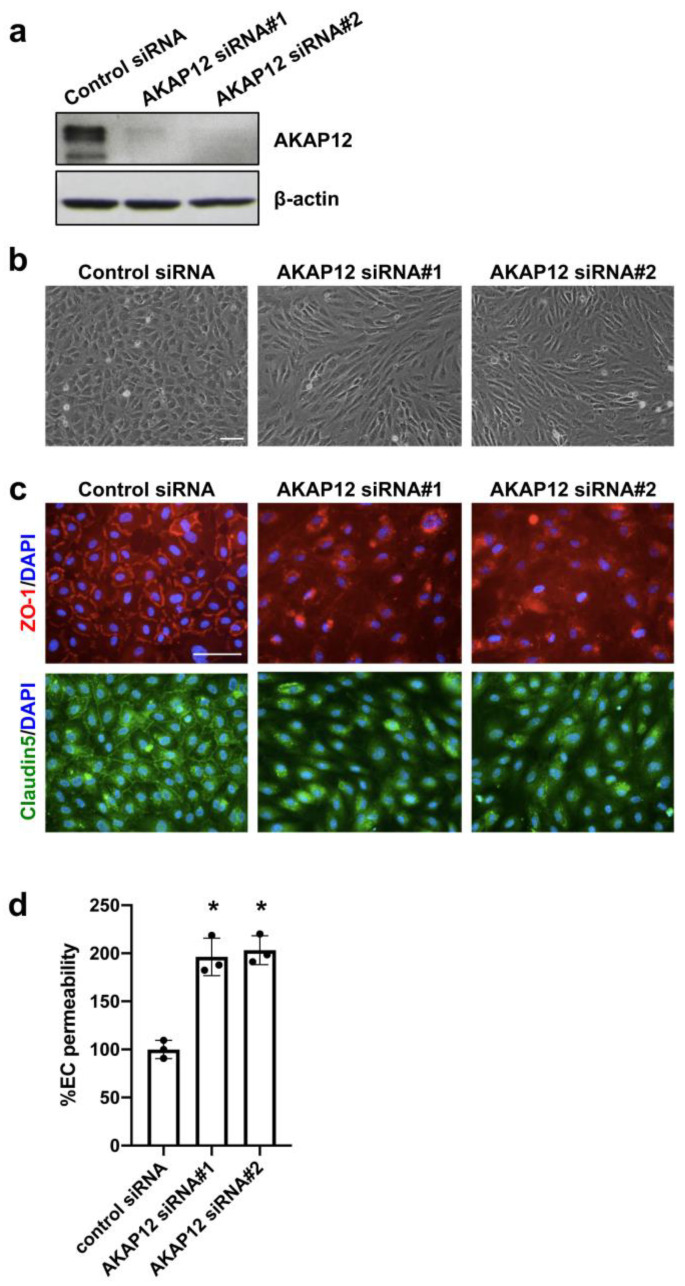
**AKAP12 and in vitro endothelial permeability:** (**a**) In cultured cerebral endothelial cells, two kinds of *Akap12* siRNAs (siRNA#1 and siRNA#2) suppressed AKAP12 expression. (**b**) AKAP12-deficient cells showed different morphology compared to control-siRNA-treated endothelial cells. Scale bar = 100 μm. (**c**) In addition, the expression patterns of ZO-1 (red) and Claudin-5 (green) were disrupted in AKAP12-deficient endothelial cells. Scale bar = 100 μm. (**d**) Concomitantly, in vitro endothelial permeability was increased by AKAP12 downregulation. Mean ± SD of *n* = 3. * *p* < 0.05 vs. control siRNA group. One-way ANOVA followed by post-hoc Tukey’s multiple comparisons test.

We next examined the mechanisms by which AKAP12 supports BBB function. To conduct loss-of-function experiments, we used two different siRNA sequences for *Akap12*, and they both successfully downregulated AKAP12 expression in cultured endothelial cells (Figure 3a). The cell morphology and the expression patterns of ZO-1/Claudin5 in AKAP12-deficient endothelial cells were different from that of the control cells (i.e., AKAP12-expressing endothelial cells) (Figure 3b,c). Importantly, the transwell-based in-vitro endothelial permeability assay showed that AKAP12 deficiency caused a larger extent of endothelial permeability (Figure 3d), indicating that AKAP12 plays an important role in endothelial tightness.

**Figure 4:**
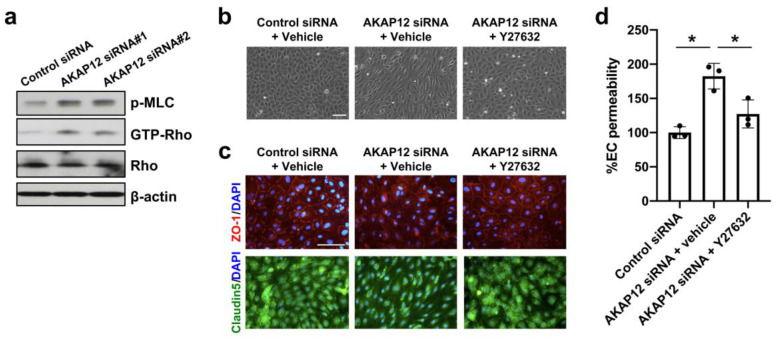
**Rho kinase and in vitro endothelial permeability:** (**a**) In cultured cerebral endothelial cells, *Akap12* siRNAs (siRNA#1 and siRNA#2) activated the Rho kinase pathway. (**b**–**c**) Rho kinase inhibitor Y-27632 (10 µM) ameliorated the morphology change and ZO-1/Claudin 5 disruption in AKAP12-deficient cells. Scale bar = 100 μm. (**d**) The increase of in vitro endothelial permeability in AKAP12-deficient cells was also relieved by Y-27632. Mean ± SD of *n* = 3. * *p* < 0.05. One-way ANOVA followed by post-hoc Tukey’s multiple comparisons test.

**Figure 5:**
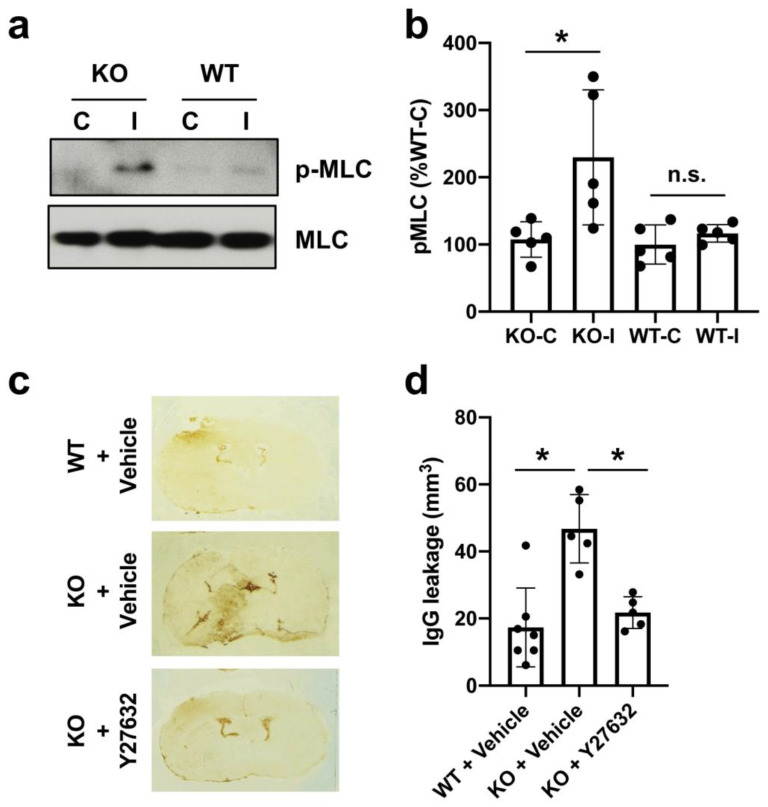
**Rho kinase and BBB damage in stroke mice:** (**a**–**b**) At 3 days after stroke, the phospho-MLC levels of brain homogenates from wild-type (WT) and AKAP12 knockout mice (KO) were analyzed by western blots. Densitometric quantification of western blot data showed that elevation of phospho-MLC levels was higher in AKAP12 knockout mice (KO) than wild-type mice (WT). Mean ± SD of *n* = 3. * *p* < 0.05. One-way ANOVA followed by post-hoc Tukey’s multiple comparisons test. C: contralateral hemisphere, I: ipsilateral hemisphere, n.s.: not significant. (**c**–**d**) IgG staining showed that treatment with Y-27632 (10 mg/Kg) rescued MCAO-induced BBB breakdown in AKAP12 knockout mice (KO). Mean ± SD of *n* = 5–6 per group. * *p* < 0.05. One-way ANOVA followed by post-hoc Tukey’s multiple comparisons test.

AKAP12 has been shown to suppress the Rho signaling pathway [17,18]. Rho kinase activity is upregulated in endothelial cells after stroke [19], and RhoA/Rho-kinase inhibition was shown to alleviate Aβ42-induced BBB disruption [20]. Therefore, we next investigated the Rho signaling pathway under the conditions of AKAP12 deficiency. In cell culture conditions, AKAP12 downregulation by siRNA induced Rho activation along with increased phosphorylation of myosin light chain (MLC), which is a substrate of Rho kinase [21] (Figure 4a). When the Rho pathway was suppressed by Rho kinase inhibitor Y-27632 (10 µM), the morphology of AKAP12-deficient endothelial cells returned to normal conditions (Figure 4b). In addition, Y-27632 treatment ameliorated the dysregulation of ZO-1/Claudin 5 expressions and the increase of endothelial permeability in AKAP12-deficient endothelial cells (Figure 4c,d). Also in vivo, compared to wild type mice, *Akap12* knockout mice showed a larger level of MLC phosphorylation in the ipsilateral side after stroke (Figure 5a,b). In addition, the treatment of Rho kinase inhibitor Y-27632 suppressed IgG leakage in *Akap12* knockout stroke mice (Figure 5c–e), which was consistent with our in vitro findings that the activation of Rho signaling pathway causes endothelial leakiness under the conditions of AKAP12 downregulation.

**Figure 6:**
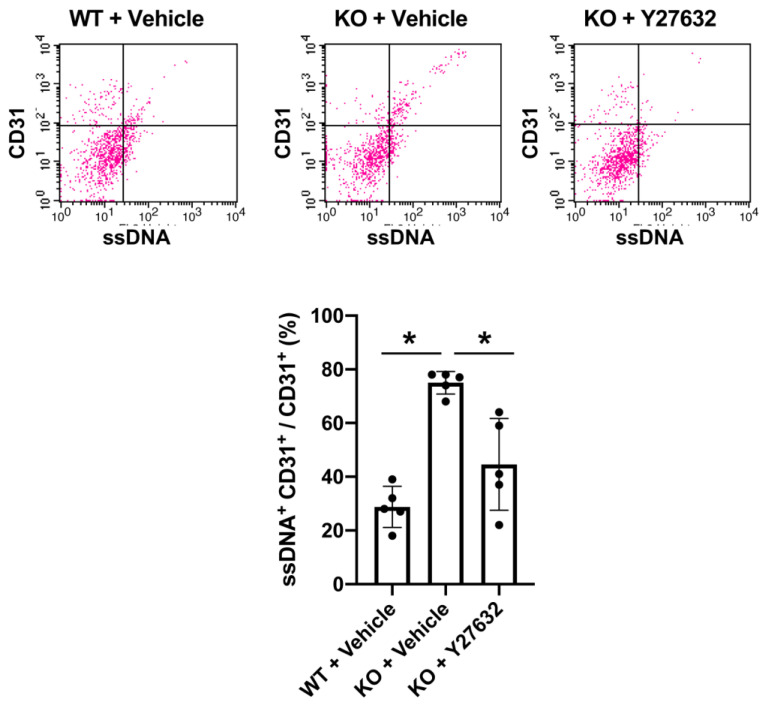
**AKAP12 and endothelial viability:***Akap12* KO mice exhibited more ssDNA-positive CD31 cells (damaged endothelial cells) after stroke. On the other hand, Y-27632 (10 mg/Kg) ameliorated stroke-induced endothelial damage in *Akap12* KO mice. Mean ± SD of *n* = 5. * *p* < 0.05. One-way ANOVA followed by post-hoc Tukey’s multiple comparisons test.

Finally, we examined whether AKAP12 expression also participates in endothelial survival, because AKAP12 is known to regulate the pro-survival PKA-CREB pathway in the brain [10]. The FACS analysis showed that *Akap12* knockout mice exhibited larger numbers of ssDNA-positive CD31 cells (i.e., damaged/dead endothelial cells) after stroke (Figure 6). Interestingly, Y-27632 treatment suppressed the cell death/damage in endothelial cells in *Akap12* knockout mice (Figure 6), supporting the idea that the AKAP12-Rho pathway may be an effective therapeutic target for stroke.

## 3. Discussion

In this study, we used in vitro and in vivo systems to demonstrate that (i) AKAP12 is upregulated in cerebral endothelial cells after ischemic stress, (ii) AKAP12 deficiency increases endothelial permeability through the disruption of tight junction proteins, and (iii) Rho kinase signaling is upregulated by AKAP12 deficiency, which results in the exacerbation of endothelial dysfunction after stroke. Treatment options for stroke remain limited to a reperfusion approach by either tPA or mechanical thrombectomy. However, the majority of stroke patients are still ineligible for these treatments, partly because of the short time windows and/or their side-effects. Because the side effects may stem from the reperfusion injury to the BBB, a combination of effective BBB-protective therapies may increase the therapeutic window of thrombectomy approaches. Therefore, our findings that AKAP12 signaling is involved in the regulation of BBB tightness under stroke conditions provide a hint towards the development of a novel therapy to protect BBB function for stroke patients.

Brain responses after stroke onset are very complicated, but roughly speaking, they can be summarized as biphasic phenomena, i.e., tissue damage during the acute phase and tissue repair during the chronic phase [4,22,23]. Therefore, research for identifying the therapeutic targets has been mostly focused on the deleterious events during the early phase and/or beneficial events for tissue remodeling during the later phase. However, even in the early phase, the brain may initiate beneficial events as a self-protective mechanism against ischemic stress. For example, during the acute phase of stroke, some soluble factors, such as tissue inhibitor of metalloproteinase 1 (TIMP-1) and pentraxin 3 (PTX3), are secreted in and/or near the damaged area to ease the breakdown of the BBB. TIMP1 attenuates BBB disruption by inhibiting the action of matrix metalloproteinases (MMPs) on extracellular matrix, and PTX3 binds to vascular endothelial growth factor (VEGF) and suppresses VEGF-induced vascular permeability [24,25]. In addition, the endogenous mechanisms of the anti-oxidant Nrf2/HO-1/HIF-1 pathway are also activated after stroke to exert neuroprotection [26]. Our current study supports and even expands these findings by showing the BBB-supportive roles of AKAP12 during the acute phase of stroke. Because BBB damage and dysfunction are a major hallmark in stroke pathology, and because BBB tightness may be disrupted by reperfusion injury after re-canalization in stroke patients, AKAP12 signaling can be a novel and effective therapeutic target for acute stroke injury.

Although our findings demonstrate the importance of AKAP12 in BBB tightness after stroke, there are some caveats and limitations that need to be addressed in future studies. First, we focused on the roles of endothelial-AKAP12. However, BBB function is known to be regulated by multiple types of brain cells besides endothelial cells, such as astrocytes and pericytes [27,28,29,30,31]. We previously showed that astrocytes and pericytes also express AKAP12 [10,12,32]. Therefore, future studies are warranted to investigate the roles of astrocyte- and/or pericyte-AKAP12 in BBB function after stroke. For this purpose, endothelial-astrocyte-pericyte co-culture systems will be useful to further examine the roles of AKAP12 in BBB integrity under physiological and pathological conditions [33]. Second, we did not examine the roles of endothelial-AKAP12 during the chronic phase of stroke. In the neurovascular unit (NVU), mechanisms of cell-cell interactions are mediated at least partly via an exchange of soluble factors [34], and some of these NVU mediators show biphasic roles after stroke, i.e., beneficial during the acute phase but deleterious during the chronic phase, and vice versa [35,36,37]. AKAP12 is known to regulate the secretion of multiple growth factors, including VEGF, which shows biphasic roles after stroke [12,38,39]. Therefore, roles of endothelial-AKAP12 during the chronic phase of stroke should be carefully examined in the future to further our understandings of the roles of AKAP12 in stroke pathology. Third, the precise mechanism by which ischemic stroke increases the expression level of endothelial AKAP12 still remains to be elucidated. Previous studies have demonstrated the importance of HIF-1 binding sites on the promotor region of AKAP12 in hypoxia-induced AKAP12 expression in human microvascular endothelial cell line 1 (HMEC-1) [40], providing us a clue for examining the mechanism of AKAP12 upregulation after ischemic stress. On the other hand, AKAP12 is known to downregulate the level of HIF-1alpha protein by enhancing the interaction of HIF-1alpha with pVHL (von Hippel-Lindau tumor suppressor protein) and PHD2 (prolyl hydroxylase 2) [32]. Gaining an understanding of the balance between AKAP12 and HIF-1 may reveal unknown mechanisms of stroke pathology. Fourth, we showed that endothelial AKAP12 was related to the cellular distribution of tight junction proteins, but we did not investigate the changes in the phosphorylation level of tight junction proteins after ischemic stress. This is an important subject for future studies because the phosphorylation level of tight junctions was reported to regulate the integrity of BBB [41]. Furthermore, our FACS approach did not distinguish apoptotic endothelial cell death from necrotic endothelial cell death. To pursue the roles of AKAP12 in endothelial function under the conditions of stroke, future studies will be required to carefully dissect the cellular mechanisms by which AKAP12 deficiency causes these endothelial dysfunctions. Lastly, although our current study demonstrates that AKAP12 is an important endogenous molecule for suppressing deleterious cascades causing BBB damage after stroke, we need to consider how to translate this finding into clinical practice. Our experiment with a Rho kinase inhibitor Y-27632 provides a proof-of-concept that the AKAP12-Rho pathway can be a therapeutic target for stroke. Nonetheless, AKAP12 is known to regulate multiple intracellular signaling pathways besides the Rho pathway, such as Src-FAK, PKA-cAMP, PKC-RAF/MEK/ERK, and JNK-AP-1 signaling pathways [9,42]. Therefore, investigations into the downstream pathways of AKAP12 will provide a hint to determine novel therapeutic targets to develop effective drugs for stroke.

In summary, our current study demonstrates that endogenous AKAP12 works to alleviate the damage and dysfunction of the BBB during the acute phase of stroke. These data support the idea that the AKAP12 pathway may represent a novel therapeutic target for stroke.

## 4. Materials and Methods

### 4.1. Animals

All experimental procedures followed NIH guidelines and were approved by the Massachusetts General Hospital Institutional Animal Care and Use Committee (#2009N000138, 19th Aug 2009). *Akap12* KO mice of C57BL/6 background were provided from the Gelman Lab at the Roswell Park Comprehensive Cancer Center [43] and were maintained/expanded in the animal facility at the Massachusetts General Hospital. Protocols for the genotyping of *Akap12* KO mouse lines are as previously described [10].

### 4.2. Focal Stroke Model in Mice

Mice (12–14 weeks) were anesthetized with 1% isoflurane in 30%/70% oxygen/nitrous oxide, with the anesthetic dose titrated to maintain spontaneous respiration. Rectal temperatures were monitored and maintained at 37 ± 0.5 °C with a thermostat-controlled heating pad. After midline skin incision, a 7-0 nylon monofilament coated with silicon resin was introduced through a small incision into the common carotid artery [44] to occlude a right middle cerebral artery (MCA). Cerebral blood flow around the territory of MCA was monitored by laser Doppler flowmetry during surgery to evaluate the severity of ischemia. Then, 45 min after MCA occlusion, the filament was withdrawn for reperfusion.

### 4.3. Cell Culture

Primary human brain microvascular endothelial cells (HBMECs) were purchased from Cell Systems (Kirkland, WA, USA) and cultured in EBM^TM^ Basal Medium (Lonza, Basel, Switzerland) with EGM^TM^ SingleQuots^TM^ Supplement Pack (Lonza, Basel, Switzerland) and 100 units/mL penicillin and 100 mg/mL streptomycin. Cells were maintained at 37 °C in a humidified atmosphere containing 5% CO_2_. For AKAP12 knockdown, HBMECs were transiently transfected with 10 nM AKAP12 siRNAs using RNAiMax (Thermo Fisher, Waltham, MA, USA) according to the manufacturer’s instructions.

### 4.4. In Vitro Stroke Model

The oxygen-glucose deprivation (OGD) system was used as an in vitro stroke model in our HBMECs. OGD experiments were conducted using a humidified anaerobic chamber (Heidolph, incubator 1000, Brinkmann Instruments, Westbury, NY, USA) kept at 37 °C, which contained an anaerobic gas mixture (90% N_2_, 5% H_2_, and 5% CO_2_). For OGD conditions, cells were maintained with glucose-free Dulbecco’s modified Eagle medium (Thermo Fisher, Waltham, MA, USA) in the anaerobic chamber. Then, 4 h later, the culture medium was replaced with the standard culture media containing glucose, and cells were moved to a regular CO_2_ incubator.

### 4.5. In Vitro Endothelial Permeability Assay

Permeability across the endothelial cell monolayer was measured by using type I collagen–coated transwell units (6.5 mm diameter, 3.0 μm pore size polycarbonate filter; Corning) [45]. After HBMECs became confluent on the Transwell, fluorescein isothiocyanate–labeled dextran (molecular weight, 40,000) was added to the upper chamber. After incubation for 30 min, 100 μL of sample from the lower compartment was measured for fluorescence at 620 nm when excited at 590 nm with a spectrophotometer.

### 4.6. Y-27632 Treatment

We prepared 10 mM Y-27632 stock solution in DMSO. Then, the stock solution was diluted by PBS as follows: 10 µM Y-27632 for in vitro experiments and 10 mg/kg Y-27632 for in vivo experiments.

### 4.7. Western Blot

Proteins were extracted from HBMECs or mouse brain tissues using Pro-PREPTM Protein Extraction Solution (Boca Scientific, Westwood, MA, USA). Lysates (20 µg per sample) with equal volumes of Novex^TM^ SDS sample buffer (Thermo Fisher, Waltham, MA, USA) and 2-ME were heated at 95 °C for 5 min and then loaded onto 4–20% Tris–glycine gels. After electrophoresis and transferring to polyvinylidene difluoride membranes (Thermo Fisher, Waltham, MA, USA), the membranes were blocked in Brock Ace (Bio-Rad, Hercules, CA, USA) for 15 min at room temperature. Membranes were then incubated overnight at 4 °C with an anti-AKAP12 antibody (1:5000, obtained from the Gelman Lab at Roswell Park Cancer Institute), phospho-MCL antibody (1:1000, Cell Signaling), Rho antibody (1:1000, Cell Signaling) or anti-β-actin antibody (1:5000, Sigma Aldrich, St. Louis, MO, USA) followed by incubation with peroxidase-conjugated secondary antibodies and detection by Pierce ECL Western Blotting Substrate (Thermo Scientific, Waltham, MA, USA).

### 4.8. Brain Microvessel Isolation

We followed the procedure in our previous study to isolate brain microvessels from mouse brains [46], which confirmed little contamination of neurons, astrocytes, and oligodendrocytes. In short, mouse brains were taken out after PBS (pH 7.4) perfusion. Cortical gray matter was dissected and rolled on filter paper to remove the large blood vessels. Tissues were then homogenized and centrifuged. The pellet was resuspended with 4 volumes of 18% dextran and centrifuged again at 1500× *g* for 20 min. The pellet was collected, and the remaining tissue was reprocessed twice in a similar fashion. All 3 pellets were lysed in lysis buffer (Cell Signaling Technology) with proteinase inhibitors for immunoblotting.

### 4.9. Rho Pull-Down Assay

Active Rho were measured using the pull-down method with Active Rho Pull-Down and Detection Kit (Thermo Fisher, Waltham, MA, USA), according to the manufacturer’s instructions. Briefly, GTP-bound Rho were pulled down by the incubation of cell lysates with GST-Rhotekin-RBD protein agarose beads, then detected by Western blot analysis.

### 4.10. Immunocytochemistry

Cells were washed with ice-cold PBS (pH 7.4), followed by 4% PFA for 15 min. After washing with PBS, cells were incubated with 3% BSA in PBS for 1 h and then incubated with primary antibody against ZO-1 (1:200; Invitrogen, Carlsbad, CA, USA) and Claudin-5 (1:200; Invitrogen, Carlsbad, CA, USA) at 4 °C overnight. After washing with PBS, they were incubated with anti-rabbit secondary antibodies conjugated with Alexa Fluor 488 or Alexa Fluor 568 (1:500; Invitrogen, Carlsbad, CA, USA) for 1 h at room temperature. Finally, nuclei were counterstained with DAPI (Electron Microscopy Sciences, Hatfield, PA, USA).

### 4.11. Immunohistochemistry

Mouse brains were taken out after perfusion with PBS (pH 7.4) and then quickly frozen using liquid nitrogen. Coronal sections of 16-μm thickness were cut on a cryostat at −20 °C and collected on glass slides. Sections were fixed by 4% PFA and rinsed 3 times in PBS (pH 7.4). After blocking with 3% BSA, sections were incubated at 4 °C overnight in PBS/0.1% Tween/0.3% BSA solution containing primary antibodies against CD31 (1:100, BD Bioscience, San Jose, CA, USA) and AKAP12 (1:500, obtained from the Gelman Lab at Roswell Park Cancer Institute). Then, sections were washed and incubated with secondary antibodies with fluorescence conjugations at room temperature for 1 h. For IgG staining, coronal sections (1 mm apart) were obtained from the brain tissues including infarct area. After PFA fixation, sections were incubated in 3% H_2_O_2_, followed by blocking with 10% BSA. The sections were then incubated overnight at 4 °C with antibody against donkey anti-mouse IgG (1:200; Jackson Immunoresearch Laboratories, West Grove, PA, USA). Immunoreactivity was visualized using the avidin–biotin complex method (ABC Staining Kits; Pierce Biotechnology, Waltham, MA, USA). The area stained with IgG was quantified using a computer-based image analysis system (Image J 1.45, Wayne Rasband, National Institutes of Health, Bethesda, MD, USA).

### 4.12. Fluorescence-Activated Cell Sorting (FACS)

Brain tissues from the peri-infarct cortex were minced and then digested at 37 °C for 30 min with an enzyme mixture (Collagenase type I, DNase I; Sigma-Aldrich, St. Louis, MO, USA). Single-cell suspensions were prepared using filtering through a 40-μm strainer. Cell suspensions were blocked with 3% BSA and then incubated with the primary antibodies against CD31 (BD Bioscience, San Jose, CA, USA) and single-stranded DNA (Merck Millipore, Burlington, MA, USA). Fluorescent-tagged Fab-specific secondary antibodies from Jackson Laboratories were incubated for 30 min at room temperature. Labeled cell populations were measured by FACSCalibur (BD Biosciences, San Jose, CA, USA). FACS data were analyzed by Cellquest pro software (BD Biosciences, San Jose, CA, USA).

### 4.13. Data Analysis and Statistics

Experiments were conducted in a blind manner. Statistical analyses were performed with Prism 8 (Graphpad, San Diego, CA, USA). To compare between two groups, statistical significance was evaluated using the Unpaired *t*-test with Welch’s correction for groups of normal distribution and the Mann-Whitney U test for groups of non-normal distribution. For multiple comparisons, the 1-way or 2-way ANOVA (for groups of normal distribution) or Kruskal–Wallis (for groups of non-normal distribution) with post-hoc test was used to evaluate significance differences. Data are expressed as mean plus and/or minus S.D. A *p* value of < 0.05 was considered statistically significant.

## Abbreviations

AKAP12	A-kinase anchor protein 12
Ang-1	Angiopoetin-1
BBB	Blood-brain barrier
HBMECs	Human brain microvascular endothelial cells
HMEC-1	Human microvascular endothelial cell line 1
NVU	Neurovascular unit
MCA	Middle cerebral artery
MLC	Myosin light chain
OGD	Oxygen-glucose deprivation
PKA	Protein kinase A
PKC	Protein kinase C
PTX3	Pentraxin 3
TIMP-1	Tissue inhibitor of metalloproteinase 1
